# The role of low glycemic index and load diets in medical nutrition therapy for type 2 diabetes: an update

**DOI:** 10.1007/s42000-024-00566-7

**Published:** 2024-05-16

**Authors:** Eleni Gerontiti, Almog Shalit, Katerina Stefanaki, Paraskevi Kazakou, Dimitrios S. Karagiannakis, Melpomeni Peppa, Theodora Psaltopoulou, Stavroula A. Paschou

**Affiliations:** 1 Endocrine Unit and Diabetes Center, Department of Clinical Therapeutics, Alexandra Hospital, School of Medicine, National and Kapodistrian University of Athens, Athens, Greece; 2 Section of Integrative Physiology and Metabolism, Joslin Diabetes Center, Harvard Medical School, Boston, MA USA; 3 Academic Department of Gastroenterology, Laiko Hospital, School of Medicine, National and Kapodistrian University of Athens, Athens, Greece; 4 Endocrine Unit, Second, Propaedeutic Department of Internal Medicine, Research Institute and Diabetes Center, Attikon University Hospital, Athens, Greece; 5 Third Department of Internal Medicine, Sotiria General Hospital, School of Medicine, National and Kapodistrian University of Athens, Athens, Greece

**Keywords:** Type 2 diabetes mellitus, Low glycemic load diets, Glycemic index, Glycemic control, Cardiovascular risk

## Abstract

The increasing prevalence of type 2 diabetes mellitus (T2DM) and its microvascular and macrovascular complications necessitate an optimal approach to prevention and management. Medical nutrition therapy serves as the cornerstone of diabetes care, reducing reliance on diabetic medications for glycemic control and mitigating cardiovascular risk. The broadening field of research in the effect of low glycemic index (GI) and/or glycemic load (GL) diets on individuals with T2DM has yielded promising results in the existing literature. Adopting low-GI and GL dietary patterns contributes to minimizing fluctuations in blood glucose levels, thus presenting a good strategy for achieving enhanced glycemic control. Furthermore, the above dietary practices may offer a viable alternative and practical approach to weight management in individuals with T2DM. However, clinical practice guidelines for diabetes dietary management show inconsistency regarding the certainty of evidence supporting the implementation of low-GI/GL nutritional patterns. This review aims to thoroughly evaluate the available data on the effectiveness of low-GI and low-GL diets in managing glycemic control and reducing cardiovascular risk factors.

## Introduction

The rising incidence of diabetes mellitus and its significant implications for the cardiovascular system have aroused alarm worldwide. The prevalence of diabetes surged from 108 million in 1980 to 536.6 million in 2021, marking the most rapid ascent in low- and middle-income countries [1, 2]. Poorly controlled diabetes mellitus can lead to long-term complications such as retinopathy, nephropathy, neuropathy, and cardiovascular disease as well as an increased risk of mortality. Notably, 43% of deaths linked to high blood glucose occur prematurely, particularly between the ages of 20 and 69 [1]. Type 2 diabetes mellitus (T2DM) is the most prevalent form of diabetes, accounting for around 90% of all cases. T2DM is caused by impaired insulin secretion by pancreatic β-cells and inadequate responsiveness of insulin-sensitive tissues to insulin, resulting in an imbalance in glucose metabolism [3]. Therefore, medical nutrition therapy (MNT) is considered fundamental to the therapeutic approach to T2DM. However, what constitutes the optimal dietary strategy remains controversial [4]. According to a recent consensus report issued by the American Diabetes Association (ADA) and the European Association for the Study of Diabetes (EASD), the efficacy of MNT in glycemic control is grounded in two key factors, namely, dietary quality and energy restriction [5]. The existing guidelines for T2DM universally support such fundamental principles as reduced calorie intake for overweight and obese individuals, replacement of saturated fats with unsaturated fats, achieving dietary fiber intake equal to or higher than those recommended for the general population, and avoiding added sugars [1]. However, the guidelines do not provide specific recommendations regarding the quality of carbohydrates, which directly impact blood glucose levels.

Available carbohydrates, such as starches and sugars, can be broken down in the intestines into simple sugars, while unavailable fibers resist digestion by human enzymes [6]. The glycemic index (GI) evaluates the quality of carbohydrate-containing foods by assessing their impact on blood glucose levels. It is calculated as a ratio of the postprandial change in blood glucose concentration (glycemic response) after consumption of a standard food portion containing 50 g of available carbohydrates relative to the glycemic response induced by 50 g of a reference carbohydrate, typically glucose or white wheat bread. The International Organization for Standardization (ISO) defines the GI on either the glucose or the bread scale. Foods containing carbohydrates that undergo rapid digestion, absorption, and metabolism are classified as high-GI foods (GI ≥ 70 on the glucose scale). In contrast, those undergoing slow digestion, absorption, and metabolism are categorized as low-GI foods (GI ≤ 55 on the glucose scale). The GI is designed for high-carbohydrate foods, but proves inadequate for evaluating mixed meals. The glycemic impact of a mixed meal is further affected by factors such as cooking and processing methods as well as the overall macronutrient composition of the diet. For instance, increasing fiber intake reduces a diet’s GI by decelerating the carbohydrate absorption rate. The glycemic load (GL) reflects the quality and quantity of consumed carbohydrates. It is calculated by multiplying the GI by the total available carbohydrate content in a specific amount of food [6]. GL is categorized as low (< 10), intermediate (11–19), or high (> 20) [7]. Most published reviews, meta-analyses, and randomized controlled trials (RCTs) investigate the effect of a low-GI diet on glycemic control, while publications addressing the impact of GL are extremely limited. In certain studies, GL was computed by multiplying the average GI of the diet by the mean daily available carbohydrate and then dividing the result by 100.

In recent years, there has been growing interest in incorporating GI and GL into dietary approaches for various diseases, including cancer, polycystic ovary syndrome, and gestational diabetes mellitus, which share insulin resistance as an underlying pathogenetic mechanism [8–10]. There is also a broadening research field and ever-increasing clinical interest in the effects of low-GI and low-GL dietary patterns on individuals with T2DM, and the literature on GI and GL diets in individuals with T2DM is showing promising results. Still, isolating the independent effects of GI and GL on glycemic control and other cardiometabolic risk factors remains challenging. However, clinical practice guidelines regarding implementation of low-GI/GL dietary therapy vary. The National Institute for Health and Care Excellence (NICE) recommends that adults with T2DM choose high-fiber, low-glycemic-index sources of carbohydrates, such as fruit, vegetables, whole grains, and pulses [11], while, on the other hand, the latest ADA annual update on “Standards of Care in Diabetes” does not provide specific recommendations for MNT related to GI or GL [12]. In the recent evidence-based guidelines issued by the Diabetes and Nutrition Study Group (DNSG) of the European Association for the Study of Diabetes (EASD), the recommendation for low-GI or low-GL diets reports moderate certainty of evidence [13]. Nonetheless, according to the Diabetes Canada Clinical Practice Guidelines, individuals with T2DM are advised to choose low-GI carbohydrate sources to improve glycemic control [Grade B, Level 2], reduce LDL-C levels [Grade C, Level 3], and decrease cardiovascular risk [Grade D, Level 4] [14].

As the GI/GL diet is not yet included in the guidelines for managing T2DM and more research is needed to determine its impact on glycemic control and reducing the risk of cardiovascular disease in people with T2DM, we conducted a review to address these issues. We searched the recent literature to gather data on GI/GL diets in T2DM patients and present their potential effects on glycated hemoglobin levels (HbA1c), postprandial glucose (PPG), fasting plasma glucose (FPG), and cardiovascular risks, such as obesity, blood lipids, blood pressure, and inflammatory markers, compared to other dietary approaches.

## Materials and methods

Although this is not a systematic review, we conducted a systematic literature search to answer the critical questions related to the topic. Specifically, we searched for RCTs on low-GI/GL diets and T2DM in English published in the PubMed database and the Cochrane Library over the last few years (up to December 2023). The following combinations of search terms were used: “low glycemic index” OR “low glycemic load” AND “type 2 diabetes” OR “diabetes.” We included studies involving adult men and non-pregnant women diagnosed with type 2 diabetes mellitus. We excluded studies conducted in non-adult patients or those focused solely on the quantity of dietary carbohydrates or where low-GI and GL diets were not the primary nutritional intervention. We also performed a manual search in reference lists of the included systematic reviews and incorporated articles that met the inclusion criteria.

## Results

The results are outlined within separate sections covering glycemic control and additional cardiovascular risk factors, including obesity, blood lipids, blood pressure, and concentrations of inflammatory biomarkers. Table 1 presents the characteristics of the included RCTs, while Table 2 highlights the statistically significant outcomes.

**Table 1:** Characteristics of included randomized controlled trials (RCTs)

Study	Duration (type of RCT)	Sample size (completed the follow-up)	Intervention	Control	Outcomes
Cox et al. (2020)[15]	Three months (parallel)	178(159)	Minimizing postprandial glucose excursions through a low-GL diet	Conventional weight-loss therapy	HbA_1c_, BL, BMI, MES^a^, physical activity, nutrition, depression, diabetes empowerment, and distress
Fabricatore et al. (2011) [16]	40 weeks (parallel)	79(50)	Low-GL diet	Low-fat diet	HbA_1c_, body weight, change in diabetes medication, WC, FPG, FINS, HOMA-IR^b^, TC, LDL, HDL, TG, SBP, DBP, hs-CRP, c-peptide
Jimenez-Cruz et al. (2006) [17]	Six weeks (crossover, 6-week washout period)	36(14)	Low-GI/GL diet	High-GI/GL diet	HbA_1c_, FPG, BMI, body weight, TC, LDL, HDL, Triacylglycerol
Nisak et al. (2010) [18]	12 weeks (parallel)	104(100)	Low-GI/GL diet	CCE dietary advice	Nutrient intake date (energy, protein, fat, carbohydrate intake and daily distribution, cholesterol, crude fiber calcium, sodium, GI, GL), HbA_1c_
Turner-McGrievy et al. (2011) [19]	22 weeks (parallel)	99(88)	Low-GI vegan diet	Low-GL ADA diet	HbA_1c_, body weight
Wang et al. (2015) [20]	12 months (secondary analysis)	252(238)	Low-GI/GL, ↓sodium, ↓saturated fat, and ↑fiber diet	Usual care	HbA_1c_, FPG, TC, LDL, HDL, TG, TC: HDL ratio, body weight, WC, BMI, SBP, DBP
Brand et al. (1991) [21]	12 weeks (crossover, 3-week washout period)	16(16)	Low-GI diet	High-GI diet	Body weight, HbA_1c_, FPG, urinary glucose, FINS, c-peptide, TC, VLDL, LDL, HDL, TG, 8-h monitoring of PPG, postprandial insulin, C-peptide, and triglycerides in high- and low-GI profile days^c^
Wolever et al. (2008) [22]	12 months (parallel)	162(156)	High-CHO, low-GI diet	1.high-CHO, high-GI diet and 2. low-CHO, high-monounsaturated-fat diet	Body weight, WC, HbA_1c_, FPG, FINS, 2-h post-OGTT glucose and insulin, FFA, TC, LDL, HDL, TG, TC: HDL ratio, Apo A1, Apo B100, Apo B: A, CRP, SBP, DBP
Cai et al. (2017) [23]	6 months (parallel)	130(130)	High-fiber, low-GI diet	Diabetes diet, exercise therapy, oral hypoglycemic drugs	Structure of intestinal flora, HbA_1c_, FPG, FINS, HOMA-IR, hs-CRP, IL-1β, IL-6
Yusof et al. (2009) [24]	12 weeks (parallel)	104(100)	Low-GI diet	CCE	HbA_1c_, Fructosamine, FPG, PPG, FINS, HOMA-IR, Body weight, BMI, WC, TC, LDL, HDL, TG, SBP, DBP
Rizkalla et al. (2004) [25]	4 weeks (crossover, 4-week washout period)	12(12)	Low-GI diet	High-GI diet	Body weight, HbA_1c_, FPG, FINS, Morning peak of glucose and insulin, 8-h AUC of glucose and insulin, TC, LDL, HDL, TG, FFA, Apo B, total fat mass, lean mass, PAI-1 activity
Pavithran et al. (2020) [26]	24 weeks (parallel)	40(36)	Low-GI diet	Usual diet	Body weight, BMI, WC, TSF, HbA_1c_, TC, LDL, HDL, TG, VLDL, total mass, fat mass, lean mass, fat-free mass, regional fat, truncal mass, fat and lean, android fat, gynoid fat, A/G ratio
Visek et al.(2014) [27]	Three months (crossover, 3-month washout period)	20(20)	Low-GI diet	Standard diabetic diet	Body mass, BMI, fat mass, fat-free mass, body cell mass, LDL, HDL, TG, HbA_1c_, FPG, insulin sensitivity, basal hepatic glucose production
Jenkins et al. (2008) [28]	Six months (parallel)	210(155)	Low-GI diet	High-cereal fiber diet	Body weight, HbA_1c_, FPG, TC, LDL, HDL, TG, TC: HDL ratio, LDL: HDL ratio, CRP, SBP, DBP, adverse Events, change in diabetes medication, satiety rating
Ma et al. (2008) [29]	12 months (parallel)	40(35)	Low-GI diet	ADA diet	HbA_1c_, TC, LDL, HDL, TG, DBP, SBP, body weight, WC

Abbreviations: GL: glycemic load; HbA_1c_: glycated hemoglobin levels (%); BL: blood lipids (mg/dl or mmol/L), BMI: body mass index (kg/m^2^); MES: medication effect score; WC: waist circumference (cm); FPG: fasting plasma glucose (mg/dl or mmol/l); FINS: fasting insulin (U/L); HOMA-IR: homeostasis model assessment of insulin resistance; TC: total cholesterol (mg/dl or mmol/l); LDL: low-density lipoprotein cholesterol (mg/dl or mmol/l); HDL: high-density lipoprotein cholesterol (mg/dl or mmol/l); TG: triglycerides (mg/dl or mmol/l); SBP: systolic blood pressure (mmHg); DBP: diastolic blood pressure (mmHg); hs-CRP: high sensitivity C- reactive protein (mg/l); GI: glycemic index; CCE: conventional carbohydrate exchange; ADA diet: diet adhering to the American Diabetes Association (ADA) guidelines; VLDL: very low-density lipoprotein cholesterol (mg/dl or mmol/l), CHO: carbohydrates; OGTT: oral-glucose-tolerance test; FFA: free fatty acid; Apo: apolipoprotein; IL-1β: interleukin-1 beta; IL-6: interleukin-6;; CCE: conventional carbohydrate exchange dietary; AUC: areas under the curve; PAI-1: plasminogen activator inhibitor-1; TSF: triceps skinfold thickness; A/G ratio: android-gynoid-ratio.

^a^The medication effect score (MES) is an index for evaluating differences in the diabetes medication, both at the baseline and post-intervention.

^b^The homeostasis model assessment of insulin resistance (HOMA-IR) is calculated as fasting plasma glucose multiplied by fasting insulin divided by 22.5.

^c^At the end of each dietary period, participants experienced a day with identical high-GI meals (high-GI profile day). Similarly, at the end of the low-GI period, a day with standardized low-GI meals was also conducted (low-GI profile day).

**Table 2:** Statistically significant differences between the intervention and control group at the end of the intervention

Study	Intervention	Control	HbA_1C_	FPG	PPG	TC	LDL	HDL	TG	Adiposity	BP
Cox et al.(2020) [15]	GEM	WL	↓			↔	↔	↔ ^a^	↔	↓BMI	
Fabricatore et al. (2011) [16]	Low-GL diet	Low-fat diet	↓	↔		↔	↔	↔	↔	↔ weight	↔ SBP ↔ DBP
Jimenez-Cruz et al. (2006) [17]	Low-GL diet	High-GL diet	↓	↔		↔	↔	↔	↔	↔ ^b^ BMI ↔ weight	
Nisak et al. (2010) [18]	Low-GL diet	CCE	↔								
Turner-McGrievy et al. (2011) [19]	Low-GI vegan diet	Low-GL ADA diet	↔							↓weight	
Wang et al. (2015) [20]	Low-GI diet	Usual care	↓	↔		↔	↔	↔	↔	↔ BMI ↓WC	↔ MAP
Brand et al. (1991) [21]	Low-GI diet	High-GI diet	↓	↔	↓	↔	↔	↔	↔	↔ weight	
Wolever et al. (2008) [22]	Low-GI diet	High-GI and low-CHO diet	↔ ^c^	↑^d^	↓	↔	↔	↓	↑	↔ weight ↔ WC	↔ SBP ↔ DBP
Cai et al. (2017) [23]	High-fiber, low-GI diet	Diabetes diet, exercise therapy, oral hypoglycemic drugs	↓	↓							
Yusof et al. (2009) [24]	Low-GI diet	CCE	↔	↔ ^e^	↓	↔	↔	↔ ^a^	↔	↔ weight ↔ BMI ↓WC	↔ SBP ↔ DBP
Rizkalla et al. (2004) [25]	Low-GI diet	High-GI diet	↓	↓	↓	↓	↓	↔	↔	↔ weight ↔ total fat mass, ↔ lean mass	
Pavithran et al. (2020) [26]	Low-GI diet	Usual diet	↓			↔	↔	↔	↔	↓weight, ↓BMI, ↓TSF, ↔ WC, ↓total, regional and truncal fat mass, ↓android and gynoid fat	
Visek et al.(2014) [27]	Low-GI diet	Standard diabetic diet	↔	↔			↔	↔	↔	↓body mass, ↓BMI, ↓fat mass	
Jenkins et al. (2008) [28]	Low-GI diet	High–cereal fiber diet	↓	↓		↔	↔	↑	↔	↔ weight	↔ SBP ↔ DBP
Ma et al. (2008) [29]	Low-GI diet	ADA diet	↔			↔	?^f^	↔	↔	↔ weight ↔ WC	↔ SBP ↔ DBP

Abbreviations: HbA1c: glycated hemoglobin levels; FPG: fasting plasma glucose; PPG: postprandial glucose; TC: total cholesterol; LDL: low-density lipoprotein; HDL: high-density lipoprotein; TG: triglycerides; BP: blood pressure; SBP: systolic blood pressure; DBP: diastolic blood pressure; ↓: a statistically significant decrease in the intervention group compared with the control group; ↔ : no statistically significant difference between the intervention and the control group; ↑: statistically significant increase in the intervention group compared with the control group; BMI: body mass index; weight: body weight; WC: waist circumference; MAP: mean arterial pressure; GEM: glycemic excursion minimization; WL: weight loss lifestyle modifications; GL: glycemic load; CCE: conventional carbohydrate exchange dietary; GI: glycemic index; ADA diet: diet adhering to the American Diabetes Association (ADA) guidelines; low-CHO: low-carbohydrate; TSF: triceps skinfold thickness.

^a^Significant increase in HDL in both groups, but no substantial difference between them.

^b^more significant reductions in BMI after the low GI/GL diet (p = 0.054).

^c^HbA1c was higher in all groups at the study’s end than at baseline.

^d^Fasting plasma glucose was lowest in the high GI diet at 12 months.

^e^No change in fasting glycemia from baseline to week 12 in the GI group VS significant deterioration in the CCE group (Δ; 0.7 ± 0.3 mmol/l, *p* < 0.05).

^f^There is an inconsistency between the reports in the Table and the main text. Empty cells signify the absence of such measurement.

### Glycemic control

#### Glycated hemoglobin levels (HbA_1c_)

Changes in HbA_1c_ or fructosamine levels are commonly utilized as outcome measures indicative of overall glycemic control. HbA_1c_ reflects the average glucose levels over the preceding 6–12 weeks, while fructosamine levels capture glycemic control over the preceding 2–4 weeks, potentially offering a more precise measure in shorter trials [30]. Cox et al. compared the impact of a diet focusing on postprandial blood glucose excursion minimization (GEM), consisting of low-GL foods, with a conventional diet for diabetes targeting weight loss (WL) [15]. This study is based on the hypothesis that adopting a low-GL diet, in contrast to a weight-loss diet targeting adipose tissue reduction for limiting insulin resistance and improving glycemic profile, can directly reduce postprandial BG, fostering confidence in the patient, thus subsequently lowering HbA_1c_ level. The mean pre-to-follow-up reduction of HbA_1c_ was more significant in the GEM group than in the WL group (-0.95% and -0.35%, respectively, p = 0.005).

Another RCT revealed that a low-GL, calorie-restricted diet displayed superior glycemic control compared to a low-fat, isoenergetic diet in overweight and obese individuals with T2DM despite the absence of significant differences in body weight [16]. Specifically, at week 20, participants in low-GL and low-fat groups achieved reductions in HbA_1c_ of 0.7 ± 0.1% and 0.3 ± 0.1%, respectively, while at week 40, these reductions were 0.8 ± 0.2% and 0.1 ± 0.2%, respectively. The increasing difference between groups was primarily due to a rise in HbA_1c_ from week 20 until the end of the study period among those in the low-fat group rather than a sustained reduction in the low-GL group. This outcome implies that individuals following a low-fat diet may unintentionally adopt a high-carbohydrate dietary pattern that exposes them to an increased risk of elevated glycemic levels.

Nisak et al. conducted a parallel RCT examining the impact of a low-GI/GL diet on dietary quality and HbA_1c_ of Asian patients with T2DM [18]. Specifically, 104 patients were randomized into two groups that received either low-GI or conventional carbohydrate exchange dietary advice for 12 weeks. The two groups did not differ regarding their demographic background. The implemented diets primarily vary in the GI/GL of carbohydrates with no substantial difference in macronutrient composition or energy intake. After 12 weeks, both groups fulfilled the carbohydrate and fat intake recommendations. No significant difference in HbA_1c_ level was reported between the two groups from baseline to the 12-week endpoint. However, a noteworthy, statistically significant, positive association was observed between dietary GI and GL and the changes in HbA1c levels. After dividing the participants into four quartiles according to their dietary GI and GL, those in the lowest quartiles for both GI (GI < 57.95) and GL (GL < 96.31) demonstrated the most substantial reduction in HbA_1c_ levels (*p* < 0.05; r = 0.03 and *p* < 0.01; r = 0.28, respectively).

Wang et al. analyzed data from the “Latinos en Control” RCT, in which a dietary intervention involving reduced GI/GL decreased sodium and saturated fat intake and increased fiber intake, was implemented among low-income Latinos with T2DM through a group-based educational approach [20]. It was observed that participants who ranked in the lowest quartile of GI at baseline (quartile range: 46.6–58.2) exhibited significantly lower mean HbA_1c_ levels (8.2%) in comparison to participants in the other three quartiles (9.3%, 9.0%, and 9.2%, respectively, p = 0.005). A sustained positive association, even after adjustments, was noted throughout the study between GI and HbA_1c_ levels. A one-unit change in GI corresponded to a 0.3% change in HbA1c levels (95% CI: 0.00% to 0.06%, p = 0.034). A positive association between GL and HbA_1c_ levels was also observed but did not reach statistical significance (*p* = 0.076).

In a 12-week RCT, Yusof et al. compared a low-GI diet (GI group) with a conventional carbohydrate exchange diet (CCE group) among Asian individuals with T2DM [24]. After 4 weeks, a more significant decrease in fructosamine levels (*p* < 0.01) was observed in the GI group compared with the CCE group (GI: 53 ± 7 and GL: 106 ± 25 in the GI group, and GI: 64 ± 5 and GL: 135 ± 37 in the CCE group at 4 weeks). At 12 weeks, there was a decrease in HbA_1c_ levels in both groups, but no significant difference between them (GI: 57 ± 6 and GL: 108 ± 32 in the GI group, and GI: 64 ± 5 and GL: 131 ± 30 in the CCE group at 12 weeks). The diminishing gap in GI and GL of the two dietary interventions over time could explain the absence of a significant correlation between the low-GI diet and HbA_1c_ levels.

In a parallel 6-month RCT, Jenkins et al. examined the effects of low-GI diets on glycemic control and cardiovascular risk factors compared with a control group adhering to a high-cereal fiber diet [28]. At the conclusion of the intervention, the low-GI diet (mean GI: 69.6 on the bread scale and mean GL: 128.9) displayed a significant decrease in both GI and GL compared to the high-cereal fiber diet (mean GI: 83.5 on the bread scale and mean GL: 166). Surprisingly, by week 24, fiber intake increased slightly more with the low-GI diet than with the high-cereal fiber diet (18.7 g/1000 kcal and 15.7 g/1000 kcal, respectively, p < 0.001). Although both groups exhibited decreased HbA_1c_ levels, the low-GI group demonstrated a more significant reduction of -0.33% compared to the other group (95% CI, –0.48% to –0.17%, *p* < 0.001).

A few studies failed to identify any relationship between a low-GI diet and HbA_1c_ levels. Specifically, in Wolever et al.’s study, there was no significant difference in HbA_1c_ levels between the three dietary interventions consisting of a high-CHO, low-GI, a high-CHO, high-GI, and a low-carbohydrate, high-monounsaturated-fat diet [22]. After a 1-year intervention, HbA_1c_ levels rose from approximately 6.1% at baseline to 6.3% in all three groups (*p* < 0.0001). Visek et al. found no significant difference in HbA_1c_ levels between a low-GI diet group (mean GI: 49%) and a group following a standard diabetic diet (mean GI: 68%, p < 0.01) in a 3-month crossover RCT involving 20 participants [27]. Moreover, in Ma et al.’s recent RCT, comparable improvements in HbA_1c_ levels were observed in a low-GI diet group (GI: 76.64 ± 1.46 on the bread scale and GL: 119.77 ± 13.75 at 12 months) and a group adhering to a diet aligned with the ADA’s latest guidelines (GI: 80.36 ± 1.40 on the bread scale and GL: 147.98 ± 13.31 at 12 months) [29]. Despite the absence of a difference in HbA_1c_ levels, the low-GI diet significantly reduced diabetic medication use.

#### Postprandial glucose (PPG)

Postprandial glucose excursions play a substantial role in the configuration of HbA_1c_ levels and may also be an independent contributor to diabetes-related complications [31, 32]. Brand et al. conducted a crossover RCT comparing the effect of a 12-week implementation of a low-GI diet (15% lower GI than the high-GI diet, *p* < 0.01) on several variables regarding glycemic control and cardiovascular risk factors [21]. A noteworthy and statistically significant finding was the lower plasma glucose level at the end of the low-GI compared to the end of the high-GI dietary period (131 ± 21 vs. 148 ± 22 mmol · h^−1^ · L^−1^, respectively, *p* < 0.05). In another RCT, participants with optimally controlled T2DM were divided into three groups, as follows: the first group followed a high-CHO, low-GI diet, the second a high-CHO, high-GI diet, and the third a low-carbohydrate, high-monounsaturated-fat diet [22]. After 1 year of intervention, although there was no substantial difference in HbA_1c_ levels among the three groups, the 2-h post-oral-glucose-tolerance test (OGTT) plasma glucose concentrations in the low-GI group were notably lower compared to those of both the high-GI and low-carbohydrate groups. According to the results of the 75-g OGTTs at baseline and at 3, 6, and 12 months of interventions, the low-carbohydrate group initially demonstrated a more rapid decrease in plasma glucose concentrations. However, this effect was not maintained over time. In Yusof et al.’s study, a subgroup of participants underwent 3-h monitoring of blood glucose changes after consuming a high-GI standard meal at the end of the 12-week study period [24]. Those following a low-GI diet exhibited significantly lower blood glucose fluctuations at every time point (0, 60, 150, and 180 min) compared to individuals adhering to a conventional carbohydrate exchange (CCE) diet (*p* < 0.05).

#### Fasting plasma glucose (FPG)

Most RCTs failed to show any improvement in FPG after a low-GI/GL diet [16, 17, 20, 21, 27]. Of note, in a 6-month parallel RCT, a more pronounced decrease in FPG was observed after a low-GI, high-fiber dietary intervention (study group) compared to a standard diabetic diet combined with exercise therapy and oral hypoglycemic drugs (control group) (FPG: 6.13 ± 0.36 mmol/L in the study group vs. FPG: 6.52 ± 0.57 mmol/L in the control group, *p* < 0.05) [23]. There was no significant difference in FPG between the two groups at baseline (FPG: 7.45 ± 0.21 mmol/L in the study group, FPG: 7.44 ± 0.23 mmol/L in the control group). Rizkalla et al. conducted a 4-week crossover RCT comparing a low-GI with a high-GI diet [25]. The primary difference between the two dietary interventions was their calculated GI (*p* < 0.0001), while the fiber intake in the low-GI diet group was higher (34 ± 3 g/day in the low-GI diet vs. 21 ± 3 g/day in the high-GI diet, *p* < 0.0001). After the intervention, FPG was significantly lower in the low-GI diet group (FPG: 10.1 ± 0.8 mmol/L at baseline and 9.19 ± 0.7 mmol/L at 4 weeks, *p* < 0.05), while a non-statistically significant increase in FPG was observed in the high-GI diet group (FPG: 9.4 ± 0.5 mmol/L at baseline and 9.8 ± 0.6 mmol/L at 4 weeks). In their RCT, Jenkins et al. concluded that there was a more significant decrease in FPG in the low-GI diet group (mean FPG: 138.8 mg/dL at baseline and 127.7 mg/dL at week 24) compared to the high-fiber diet group (mean FPG: 141.2 mg/dL at baseline and 136.8 mg/dL at week 24) (p = 0.02) [28]. In the study of Wolever et al., FPG remained stable over time in the high-GI diet group [22]. Conversely, there was an initial reduction in FPG in both the low-GI and low-CHO diet groups, followed by an increase that surpassed the concentrations observed in the high-GI diet group by 12 months. This unexpected result underscores the need for further research, especially considering that all participants in this RCT had optimal glycemic control at baseline based on their HbA_1c_ level.

### Cardiometabolic risk factors

#### Obesity

Besides body weight and body mass index (BMI), waist circumference is commonly utilized as an indicative measure of abdominal fat mass and is correlated with the risk of cardiometabolic diseases. In Wang et al.’s study, a positive correlation between GI and waist circumference over time was observed, with a one-unit change in GI corresponding to a 0.12 cm change in waist circumference (95% CI: 0.01 to 0.23, p = 0.026) [20]. A positive but non-statistically significant association between GL and waist circumference was also noted (β = 0.04, p = 0.073).

In the study by Cox et al., participants in the group guided in autonomously lowering GL (GEM group) ultimately reduced their calorie intake. They exhibited a more substantial decrease in BMI compared to those instructed to reduce daily calorie intake (WL group) (p = 0.013) [15]. It is possible that the option of a low-GL diet, achieved through the inclusion of low-GI carbohydrates and moderation of carbohydrate intake, as opposed to exclusive focus on calorie reduction, could be a more productive and palatable approach to weight loss. Turner-McGrievy et al. examined the effect of GI and GL on weight loss and HbA_1c_ in two groups, one following a vegan diet and the other following an individualized diet recommended in the 2003 ADA dietary guidelines [19]. The ADA group showed a more significant reduction in GL (-37.4 ± 52.9 in the ADA group vs. 9.5 ± 56.2 in the vegan group, *p* < 0.001). In contrast, the vegan group exhibited a more significant decrease in GI (-5.4 ± 8.2 in the vegan group vs. -1.7 ± 8.6 in the ADA group, *p* = 0.03). Both diet groups achieved a comparable reduction in energy intake, even though calorie restriction was implemented only in the ADA group for participants with BMI > 25 kg/m^2^. Nevertheless, only the low-GI vegan diet was associated with weight loss. For each point decrease in GI, there was an estimated 0.2 kg loss in participants’ weight (p = 0.001), taking into account adjustments related to dietary and demographic variables. Weight loss emerged as the sole significant predictor of HbA_1c_ (*p* = 0.047), indicating that every kilogram of body weight lost corresponded to a 0.06-point decrease in HbA_1c_. No correlation was found between changes in GL and either weight loss or improvements in HbA_1c_.

In the Yusof et al. study, there was no substantial difference in body weight, and BMI changes from baseline to the end of the 12-week intervention between the group following a low-GI diet and that adhering to a CCE diet [24]. However, a more notable reduction in waist circumference was observed in the GI group compared to the CCE group (*p* < 0.01) both at 4 weeks (-1.88 ± 0.30 cm in the GI group vs. -0.36 ± 0.4 cm in the CCE group) and at 12 weeks (-2.35 ± 0.47 cm in the GI group vs. -0.66 ± 0.46 cm in the CCE group). Pavithran et al., in their recent study, investigated the impact of a low-GI diet on South Indians with T2DM compared with their usual diet, focusing on alterations in anthropometric measurements and body composition [26]. Following a 24-week intervention, the low-GI diet group showed a more pronounced decrease in body weight (*p* = 0.007), BMI (*p* = 0.014), and triceps skinfold thickness (p = 0.001) compared to the control group. Furthermore, there was a 5.2% reduction in total fat mass in the low-GI diet group, accompanied by a significant decrease in regional fat (p = 0.001), truncal fat (*p* = 0.001), and android fat (*p* = 0.01) and gynoid fat (*p* = 0.009), with statistically significant differences compared to the control group.

#### Blood lipids

In their study, Wolever et al. emphasized a noteworthy interaction between diet and time concerning blood lipids [22]. Specifically, the rise in triacylglycerol (TG) and the decline in high-density lipoprotein cholesterol (HDL) observed in the low-GI group at 3 months demonstrated a significant moderation from 6 months until the end of the study. Furthermore, at 3 months, the low-GI group’s total HDL cholesterol ratio was 10% higher than that in the low-carbohydrate group, but this effect vanished after 6 months. No significant differences in free fatty acids (FFA), total cholesterol (TC), and low-density lipoprotein cholesterol (LDL) were highlighted. In another RCT comparing a low-GI diet with a CCE diet, no statistically significant differences were observed between the two groups at the end of the 12-week intervention period concerning TG, TC, LDL, and HDL [24]. Both groups exhibited a significant increase in HDL (*p* < 0.05), while the trajectory of TG showed a reversed pattern in correlation with time between the two groups. In the low-GI diet group, TG initially increased at 4 weeks and then decreased at 12 weeks, contrasting with the CCE diet group. In the RCT conducted by Jenkins et al., the low-GI diet showed a notable impact on HDL levels [28]. Over the 6-month study, the low-GI diet group experienced an increase in HDL by 1.7 mg/dL (95% CI, 0.8 to 2.6 mg/dL), whereas the high-cereal fiber diet group exhibited a decrease in HDL by -0.2 mg/dL (95% CI, –0.9 to 0.5 mg/dL) (*p* = 0.005). This effect persisted even after adjusting for body weight and carbohydrate and fiber intake. No significant alterations in TC, LDL, and TG levels were observed in either group from baseline to the end of the study.

#### Blood pressure

No relationship between a low-GI/GL diet and systolic and diastolic blood pressure was identified in any RCTs [16, 20, 22, 24, 28, 29]. None of these studies considered changes in antihypertensive medications, sodium intake, or other factors that might affect blood pressure measurements.

#### Inflammation

A well-established correlation exists between vascular diabetic complications and increased oxidative stress, inflammatory response, and elevated concentrations of inflammatory biomarkers [33]. CRP is the most utilized inflammatory biomarker for evaluating the influence of low-GI/GL on inflammatory response in individuals with T2DM. The study by Wolever et al. highlighted a significant decline in CRP associated with a low-GI diet [22]. Notably, in the low-GI group (GI: 55.1 ± 0.4 and GL: 133 ± 2), CRP decreased by over 20% from baseline at 12 months. In contrast, the high-GI diet (GI: 63.2 ± 0.4 and GL: 135 ± 3) showed an initial 40% rise, followed by stabilization at a 5–20% elevation by the 12-month endpoint. In the low-carbohydrate group (GI: 59.4 ± 0.4 and GL: 110 ± 2), the average CRP levels consistently fell between those observed with low-GI and high-GI diets. In Cai et al.’s study, a 6-month dietary intervention focusing on high dietary fiber and low-GI led to a noteworthy decrease in inflammatory markers, including increased sensitivity CRP (hs-CRP), interleukin-1 beta (IL-1 beta), and interleukin-6 (IL-6) [23]. This observation contrasted with that of the control group, which adhered to a standard diabetes diet, engaged in exercise therapy, and received oral hypoglycemic drugs. The remaining two RCTs, which examined the impact of a low-GL and a low-GI diet on CRP concentration, did not reveal statistically significant changes from baseline to the studies’ conclusion [16, 28].

## Discussion

The present review aimed to examine the effect of low-GI and low-GL diets on glycemic control and other cardiovascular risk factors in patients with T2DM. Most RCTs consistently conclude that adopting a low-GI/GL diet leads to improved HbA_1c_ and postprandial glycemic control in individuals with T2DM, which was not always associated with significant differences in weight reduction. This finding aligns with the conclusions drawn from existing systematic reviews [30, 34–37]. A recent systematic review and meta-analysis revealed a significant positive linear dose–response relationship between GL and HbA_1c_ levels [37]. This correlation suggests a 0.04% reduction in HbA_1c_ units for every 10-unit decrease in GL. No significant correlation was identified between low-GI/GL diets and alterations in FPG. In the three RCTs reporting a reduction in FPG following a low-GI dietary intervention, it is noteworthy that the low-GI diet consistently involved a significantly higher fiber intake than that of the control group [23, 25, 28]. Thus, alterations in intestinal flora induced by a high-fiber diet may play a substantial role. Most studies incorporate high-fiber intake into low-Gl diets [17–20, 22–28], as low-GI foods are usually fiber-rich [38]. Therefore, isolating the effect of fiber alone from that of low-GI becomes challenging. Dismissing the efficacy of low-GI foods in managing T2DM in favor of dietary fiber alone might be inappropriate. In Chiavaroli et al.’s meta-analysis, small but statistically significant decreases in FPG were observed with low-GI/GL dietary patterns [− 0.36 mmol/L (− 0.42 to − 0.19), *p* < 0.001] [37].

According to Monnier et al.’s clinical study, in inadequately controlled diabetic patients (HbA1c > 10.2%), PPG contributes approximately 30% to the 24-h glucose area under the curve (AUC), contrasting with a 70% or higher contribution in the better-controlled patients (HbA1c < 7.3%). In the HbA1c range of 7.3 to 10.2%, PPG and FPG contributed roughly equally to overall daily hyperglycemia [31]. Concerning the impact of PPG on the incidence of cardiovascular and microvascular diabetic complications, the available literature indicates a robust positive correlation, even after adjusting for HbA_1c_ levels. An in vitro study suggests that intermittent exposure to elevated glucose levels leads to a higher apoptosis rate in cultured human umbilical vein endothelial cells than in normal or constantly high glucose conditions [39]. A consistently high glucose concentration seems to trigger metabolic variations that may activate feedback mechanisms in regulatory cell controls, partially alleviating the adverse effects of glucose toxicity. The clinical significance of these findings underscores the role of glycemic fluctuations in the pathogenesis of vascular diseases in diabetic patients. All four RCTs investigating the glycemic response to a standard high-GI meal or an oral glucose tolerance test before and after the low-GI dietary intervention showed a notable improvement from baseline to the study's conclusion, demonstrating a significant difference compared to the control group. This suggests enhanced glucose tolerance, although the underlying mechanism remains poorly understood.

Even though weight loss is predominantly associated with caloric restriction, several studies concentrating on a lower-GI diet, without specific instructions in the event of an energy deficit or with similar calorie goals as the control diet, consistently report more pronounced weight loss [15, 19, 26, 27]. Consensus among existing guidelines advocates for a modest, sustained weight loss of 5 to 10% of the initial body weight. This reduction has been shown to significantly enhance insulin sensitivity and glycemic control and positively impact hypertension and lipid profiles in individuals with type 2 diabetes. The Look AHEAD (Action for Health in Diabetes) study demonstrated that individuals who shed 5% to less than 10% of their body weight (mean ± SD: 7.25 ± 2.1 kg) exhibited higher odds of achieving a 0.5%-point reduction in HbA1c, a 5 mmHg decrease in both systolic and diastolic blood pressure, a 5 mg/dL increase in HDL cholesterol, and a 40 mg/dL decrease in triglycerides [40]. Previous research suggests that lowering the GI may increase satiety, decrease caloric intake, and result in weight loss [41]. However, a recent systematic review which analyzed data from 43 cohorts, including 1,940,968 adults, revealed no consistent differences in BMI when comparing the highest with the lowest dietary GI groups [42]. Furthermore, a meta-analysis conducted in 2019 did not find any significant difference in weight loss between the low-GI diet (with ≥ 20 or < 20 units lower than the high-GI diet) and the high-GI diet in patients with impaired glucose tolerance (IGT), type 1 diabetes, or T2DM [43]. Based on these data, a change in guidelines favoring a low-GI diet for reducing body weight could not be advocated.

Initial concerns about the impact of low-GL diets on lipid profiles were raised. The suspicion was that restricting the quality and quantity of carbohydrate intake might increase daily fat intake, potentially leading to worsened lipid profiles. On the other hand, it has been shown that a diet rich in carbs might elevate the circulating concentrations of triglycerides. A positive linear dose–response relationship (p = 0.04) between the difference in GL and triglyceride levels was reported [37]. In particular, the consumption of monosaccharides, such as glucose and fructose, has been shown to promote intestinal de novo lipogenesis and the synthesis of triglycerides and lipoprotein export in the form of chylomicrons [44]. Our findings do not reveal any substantial connection between low-GI/GL diets and blood lipid levels. Instead, they suggest an elevation in HDL levels. This is in agreement with the data presented in Chiavaroli et al.’s systematic review, which highlighted that a low-GI/GL diet promotes a significantly higher reduction in HbA1c, fasting glucose, LDL, apo B, and triglycerides but not in HDL levels [37].

## Conclusions

Adopting a low-GI and -GL diet may help minimize fluctuations in blood glucose levels. This dietary pattern may improve glycemic control and reduce the inflammatory response in people with T2DM. However, the independent effect of low-GI/GL diets on whole-body insulin sensitivity is still unclear. While the data presented in this review are promising, further well-designed, large-scale, RCTs with extended follow-up periods are required before recommending such a diet as therapy for type 2 diabetes in daily clinical practice.
